# Facilitating Exercise Engagement among Community Dwelling Stroke Survivors: Is a once Per Week Group Session Sufficient?

**DOI:** 10.3390/ijerph16234746

**Published:** 2019-11-27

**Authors:** Nor Azlin Mohd Nordin, Nor Asma Husna Yusoff, Devinder Kaur Ajit Singh

**Affiliations:** Physiotherapy Programme, Faculty of Health Sciences, Universiti Kebangsaan Malaysia, Jalan Raja Muda, Abdul Aziz, Kuala Lumpur 50300, Malaysia; husnayusoff90@yahoo.com (N.A.H.Y.); devinder@ukm.edu.my (D.K.A.S.)

**Keywords:** stroke, group exercise, physical performance

## Abstract

Although exercise is proven as an effective strategy to combat post-stroke complications and the risk of stroke recurrence, many stroke survivors fail to engage in this activity following rehabilitation. In this study, we assessed the feasibility and usefulness of a low-frequency group exercise to determine its suitability as an approach to facilitate exercise engagement among stroke survivors. Forty-one stroke survivors, mean (SD) age 59.34 (10.02) years, mean time post-stroke 17.13 (17.58) months, completed a 90 minute, once per week, group exercise supervised by therapists for 12 weeks. The exercise outcomes were measured using standardized clinical tests. We observed improvement in the group’s physical performance; balance score by 3 units (Z = −3.88, *p* < 0.001), speed of repetitive sit to stand by 3.4 s (Z = −4.69, *p* < 0.001), and walking speed by 8.22 m/min (Z = −3.25, *p* < 0.001). Scores of seven out of 14 Berg’s balance scale items increased significantly, indicating better balance ability among the survivors. In conclusion, a 12-week, once per week group exercise session seems feasible and sufficient to improve the physical performance of community dwelling stroke survivors. This exercise arrangement may be offered to stroke survivors to facilitate exercise practice following rehabilitation.

## 1. Introduction

The incidences of stroke have increased dramatically over the past two decades, parallel to the increase in the number of aging populations with lifestyle diseases [1]. On the other hand, stroke mortality rate has reduced, largely due to advances in acute medical care. As a result, the world currently witnesses an increased number of stroke survivors, with a greater demand on post-stroke care [1,2]. Malaysia, being a steadily developing nation, is experiencing a similar situation; there is a need to strategize better post-stroke care [3,4].

Stroke significantly impacts the physical functioning of survivors. Studies have shown that between 35% and 80% of stroke survivors exhibit certain forms of physical dysfunction following the onset of acute stroke [5,6]. Among the survivors measured at later stage post-stroke, more than half have difficulty in walking, and nearly one-fifth were dependent on others for activities in daily living [5,6,7]. These physical dysfunctions are often associated with the presence of post-stroke impairments, resulting in an increased energy demand and oxygen consumption to perform simple day-to-day tasks. As stroke survivors experience fatigue, they tend to avoid physical activities or exercise and succumb to a sedentary life [8,9].

Lack of exercise may subsequently place stroke survivors at risk of secondary complications, such as reduced cardiorespiratory performance, muscle weakness, loss of bone density, and the formation of thrombus due to poor blood circulation in the lower limbs [10]. Together with a poor dietary habit, low exercise participation is also shown to be associated with recurrence of stroke and further co-morbidities [10]. On the other hand, regular exercise has been proven to induce various physical and psychosocial benefits, including improved mobility, balance, cardiovascular fitness, and enhanced quality of life [11]. As such, it is important to ensure that stroke survivors participate in exercise as part of post-stroke care, particularly when the survivors have completed rehabilitation.

Although much has been documented about the benefits of exercise, exercise participation among stroke survivors remains low, with time spent sitting or lying down reported as much as 22 h per day [9]. A local study reported that more than 45% of stroke survivors have no engagement in exercise upon completion of intensive rehabilitation due to various reasons, including lack of a companion, lack of confidence, and a difficulty to access exercise facilities [6,12]. Hence, there is a need for the healthcare professionals who manage post-stroke care to identify a strategy to facilitate exercise participation among stroke survivors; this may include organizing a suitable supervised exercise program in the community setting.

In planning an exercise program for stroke survivors, the guideline by the American Council of Stroke Association recommends task-oriented exercise, organized at a moderate intensity and a frequency of at least three to five times per week [13]. However, although such exercise “prescription” may be feasible to stroke survivors in developed countries, it is impractical in developing countries with low resources like Malaysia. Therefore, a more feasible exercise arrangement is needed. In this study, we evaluated the feasibility and benefits of low-frequency (once per week), therapist-supervised, group exercise for stroke survivors with regard to gains in physical performance, namely balance, functional strength, and walking speed. These three variables were targeted because they are the most affected physical performances, as reported by 35% to 55% of chronic stroke survivors, and are associated with mobility level and quality of life [6]. Group exercise was selected for practical reasons; this exercise approach requires a low number of therapists, allows a number of stroke survivors to be engaged at once, enables the survivors to be more independent during exercise, and facilitates greater interactions among the survivors. We hypothesized that a once per week group exercise is feasible and would induce positive outcomes on the desired physical performances.

## 2. Materials and Methods

### 2.1. Study Design and Location

This was a pretest–posttest experimental study conducted at the out-patient physiotherapy unit of Universiti Kebangsaan Malaysia Medical Center in Kuala Lumpur, a heavily populated area in Malaysia. The medical center has been one of the main teaching and referral hospitals for stroke care since 2009, following the establishment of the Kuala Lumpur Regional Integrated Stroke Intervention System, which aims to improve stroke care services in the country. The out-patient physiotherapy unit offers post-stroke program for community dwelling stroke survivors.

### 2.2. Study Participants

We recruited a total of 44 chronic stroke survivors (more than 6 months post-onset), who were community-ambulatory, from a pool of stroke patients who had completed intensive rehabilitation at the medical center. Being community-ambulatory was defined as, “able to walk in the community with or without walking aids and require no manual assistance.” Excluded were stroke survivors with cognitive impairment (score <24 on the mini mental state examination), a high risk for falls (score <20 on a Berg’s balance scale), or reported medical conditions that limited exercise participation, such as unstable angina pectoris, fracture within the past six months, severe lower limb arthritis, or neurological disorders complicating stroke such as a Parkinson’s disease and polyneuropathy. We determined the sample size based on a within group difference formula; a minimum sample of 44 subjects was required to detect a 15% change in all outcomes following the exercise intervention, with a two-sided 5% significance level and a study power of 80%.

However, of this number, we excluded three survivors, two of whom withdrew from the study (one decided to undergo traditional therapy while another was re-hospitalized for lung infection) and one who became ineligible at week 4 of the study due to a new diagnosis of polyneuropathy. The remaining 41 stroke survivors (mean age 59.34 (SD = 10.02) years, mean time post-stroke 17.13 (17.58) months), who completed the trial were available for post-intervention measurement and included in the final analysis. The demography and clinical profiles of the included participants are shown in Table 1.

### 2.3. Exercise Intervention

The group exercise was supervised by two physiotherapists and conducted once per week, for 90 min per session for 12 weeks. Each session started off with a warm-up and concluded with a cool-down regimen. The exercise comprised a set of pre-selected task-oriented activities, which were organized in eight stations including two rest stations (Table 2). Each station lasted for 10 min, and the stroke survivors were asked to exercise at their own comfortable pace. The task-oriented exercise in each station was progressed with regard to level of difficulty in each week. The stroke survivors’ blood pressure and heart rates were measured before and after each exercise session as a precautionary measure.

### 2.4. Assessment of Feasibility

Feasibility of the exercise program was assessed with regard to drop-out rates, adherence to exercise program, occurrence of adverse effects, and feedback from the therapists who managed the program. Adherence to exercise was measured in term of percentage of attendance through the 12-week program (attended at least 10 out of the total 12 sessions), while occurrence of adverse effects was recorded using a checklist at the completion of each exercise session and prior to the start of the subsequent exercise session. In addition, participants were advised to contact the therapists if they experience any adverse effect during the one-week exercise interval.

### 2.5. Measurements of Outcome

Measurements of physical performance among the stroke survivors were carried out at baseline and at week 13 of the intervention by a therapist who was blinded to the study intervention. Standardized measurement tools were used, namely the Berg balance scale (BBS) and the five times sit to stand (FTSTS) and timed 10-meter walk (10MWT) tests.

BBS is a 14-item scale that quantitatively measures balance and risk of falls by direct observation [14]. The items include sitting, sit to stand, transfers, standing balance, functional reaching, stepping, trunk rotation in standing, tandem stance, one legged stance, and 360° turn. Participants were guided to perform each item following the sequence from sitting to turning in standing, with rest given in between items when required. The scoring is from 0 to 4 for each item, where 0 represents an inability to complete/attempt the task and 4 represents an ability to complete the task with maximum function. The full score is 56, where a score in the range of 0–20, 21–40, and 41–56 represents having poor, moderate, and good balance, respectively. In term of risk of falls, any scores <45 indicates a risk of falling [14]. BBS has high test-retest reliability, i.e., intraclass correlation coefficient (ICC) 0.97 to 0.98 in studies involving stroke patients [15].

The FTSTS test, in which the time taken for the subject to complete five repetitions of ‘sit to stand’ from a chair with standard height is recorded, was used to assess the functional strength of the stroke survivors. FTSTS has good test-retest reliability (ICC 0.99) in measuring sit to stand ability in people with stroke [16].

The timed 10-meter walk test, a reliable (ICC 0.95–0.99) and valid gait measurement for patients who have had strokes [17], was used to measure gait speed. Time to complete walking at a comfortable pace for a specified 10 m (from a 12-m course) is taken, and gait speed is calculated using a formula; speed (meters/minute) equals 600 divided by the time in seconds [17].

Prior to the actual measurements, the participants were given two trials to familiarize themselves with the tests. The participants then performed three attempts of each test, and the average of the three attempts was calculated.

### 2.6. Statistical Analysis

All data were analyzed descriptively using a statistical package for social sciences (SPSS) version 20.0. (IBM Corporation, Armonk, NY, USA) Data were expressed as mean ± standard deviation or median (range), with a significance level set at *p* < 0.05. The Wilcoxon signed-rank test was used to compare pre- and post-intervention group data due to the data not having been normally distributed.

## 3. Results

### 3.1. Feasibility of the Exercise Program

Out of the 44 stroke survivors who participated in the program, only two (7%) withdrew, for reasons that were not associated with the exercise intervention. No adverse effects, such as dizziness, chest pain, muscle strain, ligament sprain, or delayed onset muscle soreness were reported, and the exercise adherence was 83.3% (*n* = 35). The two therapists who supervised the program expressed the opinion that the exercise program was do-able.

### 3.2. Post-intervention Outcomes

Table 3 shows the change in group scores for balance, functional lower limb strength, and walking speed following the group exercise. The score for BBS increased from 51.00 (24–56) to 54.00 (34–56) (Z = −3.88, *p* < 0.001), while score for the FTSTS test reduced by 3.40 s (Z = −4.69, *p* < 0.001), and the walking speed improved from 53.09 (12.6–120) to 61.22 (13.6–107.14) m/min (Z = −3.25, *p* = 0.001) after 12 weeks of exercise.

Change in each BBS item following the 12-week group exercise is shown in Table 4. A significant improvement was observed in 7 of the 14 items, namely standing to sitting (*p* = 0.044), standing unsupported with the eyes closed (*p* = 0.018), turn to look behind (*p* = 0.006), turn 360 degrees (*p* = 0.017), alternating foot on step (*p* = 0.034), tandem standing (*p* = 0.001), and reaching forward (*p* = 0.006).

## 4. Discussion

In this study, we sought to evaluate the feasibility and benefits of a once per week exercise among community-dwelling stroke survivors who had completed intensive rehabilitation. We found that the 12-week, weekly group exercise is feasible, indicated by a high adherence rate of more than 80%, absence of adverse effects, and positive feedbacks from the therapists who managed the program. We hypothesized that the high participants’ adherence rate, with no reported adverse effects, is likely due to the nature of the exercise program, which was tolerable and easy to cope with as the participants exercised at their own comfortable pace. We also found positive outcomes in the physical performance of our stroke survivors following the exercise program.

Numerous studies have shown that stroke survivors present an increase in postural sway when compared with healthy adults of the same age [18,19]. This balance impairment, which commonly worsens with ageing, force the stroke survivors to reduce their participation in daily activities, hence placing them at secondary risk for a decline in functional performance and health-related quality of life [20]. Balance problems were reported by nearly two-thirds of the stroke survivors in our study. As such, improving balance is important, not only to prevent falls, but also to enable them to efficiently engage in daily activities. We observed improvements in the balance performance, as measured on the BBS, following the group exercise. Although the exercise frequency and intensity differ, our findings are in agreement with that of a study by Marigold et al. [21], who reported enhancement of the mobility and balance of chronic elderly subjects who participated in a group exercise.

The positive change in balance among stroke survivors in our study is likely due to the content of the exercise program that we implemented. The group exercise included numerous exercises for training of balance, such as reaching forward while stepping on a balance pad, throwing a ball toward a trampoline and catching the bounced ball, tapping a balloon in the air while walking, walking through a walkway with obstacles, and stepping up and down a step board. Further, the exercises were progressed for level of difficulty each week, according to the individual stroke survivor’s tolerance, to maximize its benefit. Nonetheless, it is worth mentioning that, despite reaching statistical significance, the improvement in the balance score among our study participants was less than 6 points, which is regarded as a minimal clinically important change (MCIC) value for balance ability among post-stroke individuals [22]. The rather high score of BBS at the baseline could be the reason for the non-achievement of the MCIC value for balance among our subjects.

We also observed a significant reduction in the time taken to complete FTSTS among our stroke survivors following the exercise training, which indicates an increase in lower limb strength. This result is expected because the exercise program included activities such as walking on a treadmill, sit-to-stand training, stepping up and down on a step board, and cycling using a stationary bicycle, in which major lower limb strength muscles were directly involved. The literature has documented the need for strengthening exercises to be continued by stroke patients during the chronic stage, post-stroke. The importance of implementing this type of exercise is based on the findings of a number of studies that reported the occurrence of muscle alterations following stroke such as reduction of lean muscle mass, an increase in intramuscular fat deposition, and reduced VO_2_ in the affected limbs when compared with healthy limbs [23], leading to an increase in muscle fatigue and a reduction in the speed of movement.

The effects of exercise in improving the strength and functional performance of the post-stroke affected limbs had been explained by past researchers such as Kadi et al. [24], who observed improvement in muscle contraction ability following specific exercises such as treadmill walking, and Hafer-Macko et al. [23], who documented the alteration of myosin expression that helps to minimize the loss of muscle mass and strength. However, these positive findings were contradictory to the findings of a study by Schlicht et al. [25] of 12 elderly subjects who underwent an eight-week progressive strength exercise. In this previous study, no significant change was noted (*p* > 0.05), and the authors concluded that strengthening exercise alone caused no improvement in the sit-to-stand task among elderly subjects. However, unlike the exercise intervention in this past study, the group exercise in our study consisted not only strength, but also walking and endurance training. These exercise combinations could have enhanced strength retraining and contributed to the positive results of FTSTS among our study subjects.

Walking is one of the components of daily living that illustrate the quality of life level among chronic stroke survivors [26]. In our study, more than half of the stroke survivors reported walking difficulty. Our study results of walking improvement support the findings of studies by Cramp et al. [27], Pellicer et al. [28] and Pang et al. [29], who reported an increase in the walking ability and speed of stroke patients following a group exercise program of at least twice per week for 12 weeks. We noted an increase in the walking speed by 8.22 m/min among our stroke survivors after the 12-week, once per week group exercise. This increment is meaningful as it reaches the MCIC value of 8.4 m/min for gait speed among post-stroke survivors as recommended by Perera et al. [30] and can be explained by the inclusion of various walking trainings, such as over ground walking with and without obstacles and treadmill walking [31] with or without inclination up to 7%, in the exercise programs. These exercises were also progressed in term of speed and difficulty levels, thus optimizing its effects.

Our study is subject to one main limitation. Being a one-group experimental study with no control has threatened the internal validity of our results. Due to this, our study findings have to be interpreted with caution. Despite this limitation, our study demonstrates that a once-per-week group exercise supervised by therapist seems feasible and sufficient in enhancing functional performance of the stroke survivors. This favorable result appears reassuring to stroke survivors who have a difficulty to partake in exercise sessions at their own initiative. The exercise program requires the involvement of a low number of therapists, hence it can potentially be used as a strategy for a long term exercise engagement plan for stroke survivors following individual rehabilitation. Currently, such a plan is lacking, and stroke survivors are commonly discharged with advice to continue being active and plan their own exercise program, which has been found to be ineffective [13,32].

## 5. Conclusions

We conclude that a 12-week, once-a-week group exercise seems feasible, sufficient, and beneficial in improving the physical performance of chronic stroke survivors. The exercise program may potentially be offered to stroke survivors to facilitate exercise practice following rehabilitation. Further rigorous studies with a control group, including cost analyses of such an exercise program, are, however, required to confirm this study’s finding.

## Figures and Tables

**Table 1:** Demography and clinical profiles of the study participants (*n* = 41).

**Variables**	**Mean ± SD**
Age (years)	59.34 ± 10.02
Time post-stroke (months)	17.13 ± 17.58
**Gender**	***N*** **(%)**
Male	32 (78)
Female	9 (22)
**Type of stroke**	***N*** **(%)**
Ischemic	19 (46.3)
Hemorrhagic	13 (31.7)
Non-specific	9 (21.9)
**Side of stroke**	***N*** **(%)**
Right	17 (41.5)
Left	22 (53.7)
Both sides	1 (2.4)
Non-specific	1 (2.4)
**Problems related with stroke**	***N*** **(%)**
Joint stiffness	13 (31.7)
Limbs swelling	3 (7.3)
Sensation loss	4 (9.8)
Weakness of limbs	31 (75.6)
Balance problem	29 (70.7)
Walking difficulty	24 (58.5)
Speech problem	4 (9.8)
Hand dysfunction	19 (46.3)

**Table 2:** List of exercise stations and included activities.

Exercise Station	Activities	Duration (Minutes)	Intensity	Progression
Station 1 (Strengthening)	Repetitive sit to stand Wall-squat.	5 5	Own comfortable pace, repetition and sets	Carrying weights in hand, change of surface, dual tasks
Station 2 (Standing balance)	Step up and down a step board Tapping a balloon Throwing and catching ball	3 3 4	Own comfortable pace, repetition and sets	Change of surface, include stepping, dual tasks
Station 3	Rest	10	-	-
Station 4 (Walking/Cycling)	Walking on a treadmill/cycling on a stationary bike	10	Own comfortable pace	Increase inclination/ resistance, speed, dual task
Station 5 (Seated balance)	Reaching in various directions to transfer objects Balancing on a gym-ball	5 5	Own comfortable pace, repetition and sets	Change of surface, dual tasks, use multiple weights
Station 6	Rest	10	-	-
Station 7 (Hand function)	Hand cycling Graded clipping, putty pinching.	5 5	Own comfortable pace	Increase resistance, dual tasks,
Station 8 (Walking)	Obstacles course walking Walking while maneuvering ball or tapping a balloon	5 5	Own comfortable pace	Increase obstacles, dual tasks

**Table 3:** Scores for the Berg balance scale (BBS), the five times sit to stand (FTSTS) test, and the timed 10-m walk test at baseline and post-exercise.

Measures	Median (range) at Baseline, (*n* = 41)	Median (range) at Week 12, (*n* = 41)	Wilcoxon Signed Ranks Test, Z	*p*
Total score for BBS	51.00 (24–56)	54.00 (34–56)	−3.88	<0.001
FTSTS (secs)	15.00 (8.4–48.5)	11.62 (6–43)	−4.69	<0.001
Walking speed (m/min)	53.09 (12.6–120)	61.22 (13.6–107.14)	−3.25	0.001

**Table 4:** Changes in BBS items following the 12-week group exercise.

	Mean (SD) Baseline (*n* = 41)	Mean (SD) Week 12 (*n* = 41)	Mean Changes (SD)	*p*
Sitting unsupported	4.00	4.00	-	-
Transfer	3.93	4.00	0.073 (0.264)	0.083
Sitting to standing	3.83	3.98	0.146 (0.527)	0.083
Standing to sitting	3.90	4.00	0.098 (0.300)	0.044
Standing unsupported	3.90	3.98	0.073 (0.264)	0.083
Standing unsupported, eyes closed	3.76	3.93	0.171 (0.442)	0.018
Standing unsupported, feet together	3.37	3.56	0.195 (0.928)	0.186
Pick up object	3.73	3.88	0.146 (0.478)	0.057
Turn to look behind	3.56	3.80	0.244 (0.538)	0.006
Turn 360 degrees	2.88	3.24	0.366 (0.942)	0.017
Alternate foot on step	2.83	3.20	0.366 (1.067)	0.034
Tandem standing	2.61	3.27	0.659 (1.196)	0.001
Standing on one leg	2.59	2.78	0.195 (1.054)	0.243
Reaching forward	3.20	3.56	0.366 (0.799)	0.006
